# Breakthrough reactions of iodinated and gadolinium contrast media after oral steroid premedication protocol

**DOI:** 10.1186/1471-2342-14-34

**Published:** 2014-10-06

**Authors:** Akiko Jingu, Junya Fukuda, Ayako Taketomi-Takahashi, Yoshito Tsushima

**Affiliations:** 1 Department of Diagnostic Radiology and Nuclear Medicine, Gunma University School of Medicine, 3-39-22 Showa, Maebashi, Gunma 371-8511, Japan; 2 Department of Radiology, Gunma University Hospital, Maebashi, Japan

**Keywords:** Iodinated contrast media, Gadolinium contrast media, Breakthrough reaction, Acute adverse reaction

## Abstract

**Background:**

Adverse reactions to iodinated and gadolinium contrast media are an important clinical issue. Although some guidelines have proposed oral steroid premedication protocols to prevent adverse reactions, some patients may have reactions to contrast media in spite of premedication (breakthrough reaction; BTR).

The purpose of this study was to assess the frequency, type and severity of BTR when following an oral steroid premedication protocol.

**Methods:**

All iodinated and gadolinium contrast-enhanced radiologic examinations between August 2011 and February 2013 for which the premedication protocol was applied in our institution were assessed for BTRs.

**Results:**

The protocol was applied to a total of 252 examinations (153 patients, ages 15–87 years; 63 males, 90 females). Of these, 152 were for prior acute adverse reactions to contrast media, 85 were for a history of bronchial asthma, and 15 were for other reasons. There were 198 contrast enhanced CTs and 54 contrast enhanced MRIs. There were nine BTR (4.5%) for iodinated contrast media, and only one BTR (1.9%) for gadolinium contrast media: eight were mild and one was moderate. No patient who had a mild index reaction (IR) had a severe BTR.

**Conclusion:**

Incidence of BTRs when following the premedication protocol was low. This study by no means proves the efficacy of premedication, but provides some support for following a premedication protocol to improve safety of contrast-enhanced examinations when prior adverse reactions are mild, or when there is a history of asthma.

## Background

Intravenous contrast media are essential for modern medical imaging. Two of the most common intravenous contrast media are iodinated contrast media for computed tomography (CT) and angiography, and gadolinium contrast media for magnetic resonance imaging (MRI). These contrast media are relatively safe, but adverse reactions to contrast media are still an important clinical issue. Patient-related risk factors for acute adverse reactions to these contrast media have been established, i.e. 1) a history of previous acute reaction to contrast media, 2) a history of asthma, and 3) a history of allergies requiring medical treatment [1,2]. Although some guidelines have proposed oral steroid premedication protocols to prevent adverse reactions [1,2], some patients may have reactions to contrast media in spite of premedication (breakthrough reaction; BTR) [3-5]. The efficacy of premedication is still unclear, and in particular the frequency of BTR after gadolinium contrast media administration has been little reported.

In our institution, as a general rule, patients with a history of severe or moderate adverse reactions to contrast do not undergo contrast-enhanced examinations. Those with a history of mild reactions or a history of bronchial asthma will undergo contrast-enhanced examinations only when they have received oral premedication.

The purpose of this study was to assess the frequency, type and severity of BTR, following oral steroid premedication protocol.

## Methods

### Patients

All patients who underwent contrast-enhanced examinations using iodinated and gadolinium contrast media between August 2011 and February 2013 while following our institution’s premedication protocol were evaluated by retrospective chart review. Patients’ demographic data, history of adverse reactions (type and severity) to contrast media, history of asthma, history of other allergic reactions, reason for adopting premedication protocol, contrast media used, amount of contrast media, and type, severity, treatment and outcomes of BTR (if any) were collected. Institutional review board (Gunma University Graduate School of Medicine Human Research Committee) approval was obtained, and it was determined that informed patient consent was not required because of the retrospective nature of this study.

We did not include interventional radiology cases. The frequent use of additional drugs (including embolization materials, chemotherapeutic agents and sedatives), and the complexity of the procedures make it difficult to determine the cause of any adverse reactions.

All contrast-enhanced CT and MRI examinations following the premedication protocol were recorded using forms designated by our department. All adverse reactions occurring in our department were evaluated by a radiologist in charge, and the type, severity, treatment and outcome of the reactions were recorded. The type and severity of acute reactions were graded using an original grading system based on the ACR Manual on Contrast Media Version 7 [1] and ESUR Guidelines on Contrast Media Version 7 [2]. The grading system is as follows:

**Mild**: nausea, mild vomiting, urticarial rash, itching, mild laryngeal discomfort

**Moderate**: severe vomiting, severe urticarial rash, bronchospasm, facial and/or laryngeal edema, vasovagal reaction

**Severe**: hypotensive shock, respiratory arrest, cardiac arrest, convulsions.

Both index reaction (IR) (the reaction prompting the use of oral premedication) and BTR were recorded using the same grading system.

If premedication was administered according to the protocol of our department, the repeated reaction was considered a BTR. When an acute adverse reaction occurred, patients were usually observed in our hospital for at least one hour.

### Premedication protocol

Our departmental policy for the premedication protocol for contrast-enhanced examinations is based on the ACR Manual on Contrast Media Version 7 [1] and ESUR Guidelines on Contrast Media Version 7 [2]. The former does not differentiate severity and simply states history of reaction as a risk factor. The latter, on the other hand, states moderate to severe reactions as risk factors, and does not consider mild reactions an indication for premedication. Our own original premedication protocol is followed for patients with a history of mild adverse reactions, a history of bronchial asthma regardless of severity, or a history of allergies requiring medical treatment. The protocol is as follows:

1. Patients with a history of mild adverse reactions, history of bronchial asthma (regardless of severity), or a history of allergies requiring medical treatment will undergo contrast enhanced examinations if premedicated as follows:

1) Methylprednisolone (Medorol: Pfizer Co., Tokyo, Japan) 32 mg p.o. 12 and 2 hours prior to administration of contrast.

2. Patients with a history of moderate or severe adverse reactions to contrast media should not undergo contrast-enhanced examinations, with or without premedication.

2) Diphenhydramine chlorate (Restamin: Kowa Co., Tokyo, Japan) 50 mg p.o 1 hour prior to administration of contrast. This is optional, and contraindicated for glaucoma and benign prostatic hypertrophy.

Patients with a history of moderate or severe adverse reactions to contrast media are not the target of the protocol, and usually do not undergo a contrast-enhanced examination. In our hospital, the decision to perform the protocol is made by a physician who orders a radiological examination, if necessary after discussion with a radiologist.

### Contrast media

In our hospital, high-osmolality contrast media (HOCM) is no longer used, and all contrast-enhanced CT are performed using low-osmolality contrast media (LOCM).

We use four kinds of iodinated contrast media. They are Iopamidol (Iopamiron; Bayer Co. Ltd., Osaka, Japan), Iomeprol (Iomeron; Eisai Co. Ltd., Tokyo, Japan), Iohexial (Omnipaque; Daiichi-Sankyo Co. Ltd., Tokyo, Japan), and Ioversol (Optiray; Daiichi-Sankyo Co. Ltd., Tokyo, Japan).

Three kinds of gadolinium contrast media are used, which are Gadoterate meglumine (Magnescope; Terumo Co. Ltd., Tokyo, Japan), Gadteridol (ProHance; Eisai Co. Ltd., Tokyo, Japan), and Gadopentetate dimeglumine (Magnevist; Bayer Co. Ltd., Osaka, Japan). Selection of contrast media was based on the purpose of the examination and the patient’s weight. Although there are few data to support changing the contrast media to decrease the likelihood of a subsequent reaction in patients with a history of prior allergic-like reactions to iodinated contrast media, we usually change the contrast media administered in such cases.

### Data analysis

The incidence of BTR was calculated, and the type and severity of BTR were compared to those of IRs.

## Results

A total of 252 examinations using iodinated contrast media (n =198, CTs) or gadolinium contrast media (n =54, MRIs) were performed following the premedication protocol on 153 patients (63 males, 90 females, age 15–87 years) during the study period. During this time, a total of 61,810 examinations (41,388 CTs, 20,422 MRIs) were performed in our institution.

The total number of adverse reactions to iodinated contrast media during this period (with and without premedication) was 110, or 0.27% of the total number of contrast enhanced CTs. The total number of adverse reactions to gadolinium contrast media during this period was 26 or 0.13% of the total number of contrast enhanced MRIs.

### BTR of iodinated contrast media (CT)

The reasons for following the premedication protocol for 198 examinations in 125 patients using iodinated contrast media and the severity of BTRs are summarized on Tables 1 and 2. The reasons for following the premedication protocol were a history of acute adverse reactions to iodinated contrast media in 117 examinations, a history of acute adverse reactions to gadolinium contrast in six examinations, a history of asthma in 64 examinations, and suspected delayed adverse reaction to iodinated contrast media in 11 examinations. The prior adverse reactions were mild for 117 examinations, moderate for six, and severe for zero. Among these 198 examinations in 125 patients, there were BTRs in nine examinations (4.5%; 9/198 examinations, Table 3): eight were mild, one was moderate.

**Table 1:** Severity of IR and severity of BTR to iodinated contrast media (CT)

**Initial reaction (IR; n =134)**		**Breakthrough reaction (BTR)**		
		**Mild**	**Moderate**	**Severe**
Iodine	Mild (n =113)	7	1	0
	Moderate (n =4)	0	0	0
Gadolinium	Mild (n =4)	0	0	0
	Moderate (n =2)	0	0	0
Others* (n =11)		0	0	0

*Suspected delayed adverse reactions to iodinated contrast media (n =11).

**Table 2:** Severity of BTR to iodinated contrast media in premedicated asthmatic patients (CT)

	**Breakthrough reaction (BTR)**		
	**Mild**	**Moderate**	**Severe**
Asthma (n =64)	1	0	0

**Table 3:** Details of BTR of iodinated contrast media after premedication (CT)

**Pts. no.**	**Sex/age (years)**	**No. of repeat BTRs**	**BTR**		**IR**	
			**Severity**	**Nature**	**Severity**	**Nature**
1*	M/62	1	Mild	urticaria, itching	Mild	urticaria, itching
2	M/61	1	Mild	rash	Mild	nausea
3	M/67	1	Mild	mild laryngeal discomfort	-	asthma
4	F/41	1	Mild	nausea	Mild	nausea
5	F/58	2	Mild	nausea/nausea	Mild	nausea
6	M/70	1	Mild	nausea	Mild	nausea
7	F/64	1	Mild	urticaria	Mild	urticaria
8	F/67	1	Moderate	severe urticarial rash/facial edema	Mild	discomfort

*This patient presented with urticarial rash upon administration of premedication, and the rash worsened after administration of contrast.

The patient (No. 1) who experienced a mild BTR presented with urticarial rash upon administration of premedication, and the rash worsened after administration of contrast, so it was difficult to tell whether premedication or contrast was the cause of the rash.

### BTR of gadolinium contrast media

The reason for following the premedication protocol for 54 examinations in 40 patients using gadolinium contrast media, and the severity of BTRs are summarized in Tables 4 and 5. The reasons for following the premedication protocol were a history of acute adverse reactions to iodinated contrast media in 23 examinations, a history of acute adverse reactions to gadolinium contrast media in six examinations, a history of asthma in 21 examinations, and others in four examinations. The prior acute adverse reactions to gadolinium were all mild, but the prior acute adverse reactions to iodinated contrast media were moderate for two, and severe for six examinations. Among the 54 examinations using gadolinium contrast media after premedication, there was mild BTR in only one examination (1.9%; 1/54 examinations, Tables 4 and 5). This patient received premedication because of a history of bronchial asthma, and the BTR was nausea.

**Table 4:** Severity of IR and severity of BTR to gadolinium contrast media

**Initial reaction (IR; n =33)**		**Breakthrough reaction (BTR)**		
		**Mild**	**Moderate**	**Severe**
Iodine	Mild (n =15)	0	0	0
	Moderate (n =2)	0	0	0
	Severe (n =6)	0	0	0
Gadolinium	Mild (n =6)	0	0	0
Others* (n =4)		0	0	0

*In two patients, drug eruption was clinically suspected, but the causal medication was not identified. In another two patients, mild delayed reaction to iodinated contrast media was clinically suspected.

**Table 5:** Severity of BTR to gadolinium contrast media in premedicated asthmatic patients

	**Breakthrough reaction (BTR)**		
	**Mild**	**Moderate**	**Severe**
Asthma (n =21)	1	0	0

## Discussion

Acute adverse reactions to contrast media will occur at a certain rate. Non-ionic iodinated contrast is said to have an adverse reaction rate of about 0.6 to 3.1% [6-10]. This rate is said to be about 0.07 to 0.67% for gadolinium contrast media [6-9].

The ACR Manual on Contrast Media [1] and EUSR Guidelines on Contrast Media [2] recommend premedication for patients at risk for adverse reactions to contrast, but the definition of at-risk has some variation among protocols and studies. The ACR Manual states patients with a history of adverse reactions should be premedicated, but does not elaborate on the degree of the reaction. The ESUR Guidelines, on the other hand, includes patients with a history of moderate or severe reactions. When following the ESUR Guidelines, patients with history of mild reactions to contrast undergo contrast enhanced examinations without premedication. (We chose to follow and reference the older versions of the ACR Manual and ESUR Guidelines, as these were the ones available when we created our protocol and conducted our study). Our own original institutional protocol includes patients who have a history of mild adverse reactions to contrast, a history of asthma, or a history of allergies requiring medical treatment as at-risk patients requiring premedication. Patients with a history of moderate or severe reactions to contrast are advised not to undergo a contrast-enhanced study and consider an alternative examination.

Acute adverse reactions are known to occur even when the patient is premedicated. Such reactions are called BTRs [3-5,11], while the initial adverse reaction occurring without premedication is called the index reaction (IR) [3]. The term BTR has been used in the literature to indicate a reaction that occurs after contrast administration in patients who have been premedicated with corticosteroids [3-5,11]. Davenport et al. defined to this term more restrictively to refer to only those allergic-like reactions that occurred in premedicated patient with a history of prior allergic-like reactions to iodinated contrast media [3]. In our study, we used this term to refer to acute adverse reactions that occurred in a premedicated patient regardless of the indication for premedication (whether it was a history of prior acute adverse reactions to contrast media, a history of bronchial asthma, or a history of allergies requiring medical treatment).

In this study, the total rate of BTRs was 4.0%: the rate of BTRs for iodinated contrast media was 4.5%, and the rate of BTRs for gadolinium contrast media was only 1.9%. Nearly all were mild BTRs, but there was one moderate BTR. There were no patients who had a mild IR followed by a severe BTR.

Davenport et al. reported that for iodinated contrast media, the rate of BTRs was 18% [3], while Kim et al. reported a rate of 16.7% [11]. The results of our current study showed a somewhat lower rate. The reason for the lower rate of BTRs in our study is not entirely clear, but a possible explanation is the smaller percentage of examinations with a prior reaction that was moderate or severe. In the current study, only 4.2% (9/214) of iodinated contrast-enhanced examinations were performed for patients with moderate IRs for iodinated or gadolinium contrast media, and no patients in our study had severe IRs. Our current departmental policy is to premedicate patients with a history of mild adverse reactions to contrast, and to try to avoid its use entirely in patients whose prior reactions are moderate or severe. Ten percent (106/1044) and 43% (13/30) of the patients in Davenport et al. [3] and Kim et al. [11] had moderate or severe IRs. In our institution, we are frequently consulted for alternative examinations for such patients, and recommend ultrasound or MRI without contrast (or with contrast for patients whose only reason for concern is a history of adverse reactions to iodinated contrast media), or nuclear medicine examinations such as PET. Japanese physicians tend to be extremely wary of acute adverse effects to contrast enhanced examinations, and it is likely that they will avoid ordering additional contrast enhanced examinations with or without premedication for a patient with a history of adverse reactions with contrast, even when said reactions are mild. Considering this tendency, we did not expect six examinations with a history of moderate reaction to iodinated contrast and two examinations with a history of moderate reaction to gadolinium contrast media to receive contrast with premedication. Our current hospital policy is for the referring clinician to decide the indication for contrast use and also premedication. The radiology department may make recommendations to abstain from contrast use, but the patient and referring clinician make the final decision.

Another reason for our lower rate of BTRs may be our definition of BTRs. In this study, we defined BTR as acute reactions that occurred after premedication not only in patients with a prior history of reaction to contrast, but also patients with history of asthma or other allergies requiring medical treatment.

BTRs exceeded IRs in 11.1% (1/9) of iodinated contrast-enhanced examinations in our study. Freed et al. report BTRs exceeding IRs in 11% [5], and Kim et al. report 0% [11]. There have, however, been no reports of a mild IR followed by a severe BTR, and there were no such patients in our study, either.

There have been few reports on BTRs for gadolinium contrast media. Dillman et al. [9] reported nine BTRs among 78,353 examinations. In their study, however, the total number of patients undergoing premedication was unknown, thus the incidence of BTRs after premedication could not be calculated. In our study, we observed a total of 54 premedicated examinations, and there was mild BTR in only one examination (1.9%). In 23 of 54 examinations, premedication was administered because of a history of acute adverse reaction to iodinated contrast media. Although, to our knowledge, there have been no reports suggesting cross-reactivity between iodinated and gadolinium contrast media, a history of acute adverse reaction to iodinated contrast media can arguably be considered a history of allergy requiring treatment. In our study, there was no BTR in patients who were premedicated because of IRs for iodinated contrast media.

Bronchial asthma is a known risk factor for adverse reactions to contrast media [1,2,12]. In the current study, the incidence of BTR after premedication for patients with a history of bronchial asthma was low (2.4%; 2/85) regardless of severity of asthma: one patient receiving iodinated contrast media complained of mild laryngeal discomfort and another receiving gadolinium contrast media experienced nausea.

A limitation of this study was that since this was a retrospective study, there was no control group consisting of patients undergoing contrast-enhanced study without premedication. Since performing a contrast-enhanced examination without premedication on a patient with known risk factors can arguably be considered unethical, it would probably be difficult to conduct a study with a control group, even as a prospective study. This makes it very difficult to provide evidence that premedication indeed reduces the rate of adverse reactions. Another limitation of this study was that not all patients experiencing an IR underwent additional contrast enhanced examinations. Since the decision to use contrast was made by the referring physician, there may be a selection bias.

## Conclusions

The incidence of BTR after premedication was low. When prior adverse reactions are mild, or when there is a history of asthma, following a premedication protocol may make it possible to safely perform contrast-enhanced examinations. However, when IR is moderate or severe, contrast should only be used with extreme caution even with premedication.

## Competing interests

The authors declare that they have no competing interests.

## Authors’ contributions

AJ collected data and participated in study design and drafted the manuscript. JF collected data and participated in study design. ATT participated in study design and helped draft the manuscript. YT conceived the study and helped draft the manuscript. All authors read and approved the final manuscript.

## Pre-publication history

The pre-publication history for this paper can be accessed here:

http://www.biomedcentral.com/1471-2342/14/34/prepub
